# Characterization of subcellular localization of duck enteritis virus UL51 protein

**DOI:** 10.1186/1743-422X-6-92

**Published:** 2009-07-03

**Authors:** Chanjuan Shen, Yufei Guo, Anchun Cheng, Mingshu Wang, Yi Zhou, Dan Lin, Hongyi Xin, Na Zhang

**Affiliations:** 1 Avian Diseases Research Center, College of Veterinary Medicine of Sichuan Agricultural University, Ya'an, Sichuan, 625014, PR China; 2 Key Laboratory of Animal Diseases and Human Health of Sichuan Province, Ya'an, Sichuan, 625014, PR China

## Abstract

**Background:**

Knowledge of the subcellular localization of a protein can provide useful insights about its function. While the subcellular localization of many alphaherpesvirus UL51 proteins has been well characterized, little is known about where duck enteritis virus (DEV) UL51 protein (pUL51) is targeted to. Thus, in this study, we investigated the subcellular localization and distribution of DEV pUL51 by computer aided analysis, as well as indirect immunofluorescence (IIF) and transmission immunoelectron microscopy (TIEM) approaches in DEV-infected cells.

**Results:**

The DEV UL51 gene product was identified as an approximate 34 kDa protein in DEV-infected cells analyzed by western blotting. Computer aided analysis suggested that DEV pUL51 is not targeted to the mitochondrial, extra-cellular or nucleus, but be targeted to the cytoplasmic in host cells, more specifically, palmitoylation of the pUL51 through the N-terminal cysteine at position 9 makes membrane association and Golgi localization possible. Using IIF analysis, we found that DEV pUL51 was first detected in a juxtanuclear region of DEV-infected cells at 9 h postinfection (p.i.), and then was detected widely distributed in the cytoplasm and especially was stronger in the juxtanuclear region from 12 to 60 h p.i. TIEM analysis revealed that DEV pUL51 was mainly associated with cytoplasmic virions and also with some membranous structure near the pUL51-specific immuno-labeling intracellular virion in the cytoplasmic vesicles; moreover, the pUL51 efficiently accumulated in the Golgi apparatus at first, and then was sent to the plasma membrane from the Golgi by some unknown mechanism.

**Conclusion:**

In this work, we described the basic characteristics of pUL51 subcellular localization and distribution for the first time. From these results, we concluded that palmitoylation at the N-terminal cysteine, which is conserved in all alphaherpesvirus UL51 homologs, is required for its membrane association and Golgi localization, and the pUL51 mainly localized to the juxtanuclear region of DEV-infected cells, as well seemed to be incorporated into mature virions as a component of the tegument. The research will provide useful clues for DEV pUL51 functional analysis, and will be usefull for further understanding the localization properties of alphaherpesvirus UL51 homologs.

## Background

Duck enteritis virus (DEV) is a member of the subfamily *Alphaherpesvirinae*, and an important pathogen of waterfowl (ducks, geese, and swans), causing an acute contagious viral disease that result in substantial economic losses [1-3]. The genome of DEV is comprised of an approximate 180 kbp of linear and double-stranded DNA molecule, and its genomic structure is similar to that of other alphaherpesviruses [4,5]. In 2006, the DEV UL51 gene was isolated and identified from DEV CHv strain in our laboratory [6,7]. It was reported that UL51 gene of the alphaherpesviruses, which encodes a phosphorylated and palmitoylated tegument protein [8,9], and was high conserved in the alphaherpesvirus family [10]. Recent research has shown that the product of the herpes simplex virus (HSV-1) UL51 gene is a membrane associated protein, eventually incorporated into virions and forming the outer layer of tegument [9,11]; moreover, the HSV-1 UL51 protein (pUL51) appears to play multiple roles in viral replication, including egress of virus particles from the perinuclear space and secondary envelopment in the cytoplasm [12].

The infective properties of a virus are determined by the viral proteins that make up its capsid, envelope (tegument), and spikes. Although viruses are acellular organisms, viral proteins are required to reside in various cellular compartments of the host cell to fulfill their functions [13]. Therefore, knowledge of the subcellular localization of viral proteins in a host cell or virus-infected cell is very useful for in-depth studying of their functions and mechanisms as well as designing antiviral drugs. While the intracellular localization of many alphaherpesvirus UL51 proteins, such as HSV-1 [11], bovine herpesvirus 1 (BHV-1) [14], and pseudorabies virus (PrV) [15], has been well characterized, little is known about where DEV pUL51 is targeted to. In the present study, we characterized the DEV pUL51 subcellular localization by computer aided analysis, as well as indirect immunofluorescence (IIF) and transmission immunoelectron microscopy (TIEM) approaches in DEV-infected cells. There would be a strong degree of complementarity between the use of computational tools and experimental methods that can score the likelihood that DEV pUL51 belongs to a given compartment. The research will provide useful clues for DEV pUL51 functional analysis, and will be usefull for further understanding the localization properties of alphaherpesvirus UL51 homologs.

## Methods

### Computer aided analysis

The DEV UL51 gene (GenBank No. DQ072725), with a size of 759 bp, encoded a 252 amino acid protein, was identified in our laboratory [6]. Based on predicted amine acid sequence of DEV pUL51, various bioinformatics-aided tools: TargetP 1.1, SignalP 3.0 and TMHMM 2.0 server (from the search engine ) [16], PredictNLS server (from the search engine ) [17], CSS-Palm 2.0 online server (from the search engine ) [18], and Golgi predictor (from the search engine ) [19], were used to analyze the possible localization of the pUL51.

### Virus strain and cell

DEV CHv strain is a high-virulence field strain isolated from china, obtained from Key Laboratory of Animal Disease and Human Health of Sichuan Province [20,21]. Duck embryo fibroblasts (DEF) were cultured in MEM medium (Gibco-BRL) supplemented with 10% fetal bovine serum (FBS) (Gibco-BRL) at 37°C. For virus infection, MEM medium supplemented with 2–3% FBS was used.

### Antibody

A rabbit polyclonal UL51 antiserum (obtained from our Laboratory), raised against a recombinant 6-His-UL51 fusion protein expressed in *E. coli*, was purified using caprylic acid and ammonium sulfate precipitation and High-Q anion-exchange chromatography [22,23]. The purified UL51 antiserum was subsequently used as primary antibody. Besides, a pre-immune rabbit serum was also obtained from our Laboratory and purified as described above. The purified pre-immune serum was used as a negative control.

### Western blotting

DEF grown in the 6-well plates, were either mock-infected or infected with DEV CHv strain at a multiplicity of 5 PFU per cell, and harvested at 24 h p.i. Cells were lysed in SDS sample buffer, electrophoretically separated on SDS-polyacrylamide gels (SDS-PAGE) and electrically transferred to polyvinylidene difluoride (PVDF) membranes (Amersham Japan). A nonspecific protein binding was blocked by treating membranes at 4°C overnight with TBST (25 mmol Tris-HCl, 150 mmol NaCl, pH 7.4, and 0.05% Tween-20) containing 5% bovine serum albumin (BSA). Then, the membranes were incubated at 37°C for 1 h with a 1:1000 dilution of the purified UL51 antiserum or pre-immune serum in TBST containing 0.1% BSA. After washing 3 times with TBST, the membranes were incubated at 37°C for 1 h with a 1:10000 dilution of goat anti-rabbit peroxidase-labeled second antibody (Sino-American Biotechnology Co., Shanghai, China). Washed 3 times with TBST again, the membranes were subsequently treated with an enhanced chemiluminescence (ECL) western blotting detection system (Amersham) and exposed to Hyperfilm-ECL (Amersham).

### IIF

DEF, grown on coverslips in the 6-well plates, were either mock-infected or infected as described above. At different times (3, 6, 9, 12, 24, 36, 48 and 60 h p.i.), the cells were fixed with 4% paraformaldehyde for 20 min at 4°C and permeabilized with 0.1% Triton X-100 for 20 min at room temperature. The cells were then washed once with PBS and blocked for 1 h in PBS containing 10% BSA at 37°C. They were then incubated with a 1:100 dilution of the purified UL51 antiserum or pre-immune serum at 4°C overnight, washed 3 times for 10 min in PBS, and then treated with FITC conjugated goat anti-rabbit IgG (Sino-American Biotechnology Co.) for 45 minutes at 37°C. As described by Miller [24], the cell nuclei were visualized by 4',6-diamidino-2-phenylindole (DAPI) counter-staining (5 μg/ml, Beyotime Institute of Biotechnology, Shanghai, China). Fluorescent images were examined under the Bio-Rad MRC 1024 imaging system.

### TIEM

DEF were grown in the 6-well plates and were either mock-infected or infected as described above. At different times (3, 6, 9, 12, 24, 36, 48 and 60 h p.i.), the cells were fixed with modified PLP fixative (10 mM NaIO4, 75 mM lysine, 37.5 mM phosphate buffer (PB) (pH 7.4), 4% paraformaldehyde, 0.1% glutaraldehyde) for 4 h and then washed with 0.1 M PB [25]. The cells were then harvested from the 6-well plates by scraping, resuspended in PB, and pelleted by low-speed centrifugation. The cell pellet was washed with PB, dehydrated through a graded series of ethanol, and embedded in LR White resin (London Resin Company) according to the manufacturer's instructions. Ultrathin sections were collected onto Formvar-coated nickel grids. The sections were incubated with 20% normal goat serum in PBS containing 1% BSA for 1 h at room temperature, washed five times in PBS, and incubated with the purified UL51 antiserum or pre-immune serum adequately diluted in PBS-1% BSA for 2 h. After five washes in PBS, the sections were incubated for 1 h with the secondary antibody goat anti-rabbit immunoglobulin conjugated with 10-nm-diameter gold particles (British BioCell International) and then washed five times in PBS and twice in double-distilled water. The sections were double stained with 4% uranyl acetate for 30 min followed by Reynold's lead citrate solution for 5 min [11]. Carbon-coated sections were examined with a Hitachi H-600 transmission electron microscope at 75 kV.

## Results

### Subcellular localization prediction of DEV pUL51

The DEV pUL51 contains no potential mitochondrial targeting peptide, signal peptides, transmembrane helices and nuclear localization signal (NLS). However, it possesses one potential palmitoylation site at the position 9 amine acid (cysteine) near the N-terminal of the pUL51 with a high score (4.217). Moreover, the pUL51 is predicted as a Golgi type II membrane protein (Golgi localised transmembrane protein) with index values (20.662) greater than the threshold (20.005).

### Reactivity and specificity of the UL51 antiserum

**Figure 1:**
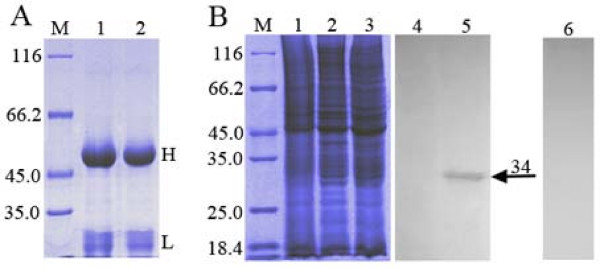
**(A) SDS-PAGE analysis of the purified UL51 antiserum and pre-immune serum**. The purified IgG proteins of UL51 antiserum and pre-immune serum were respectively examined by SDS-PAGE (lanes 1 and 2). Molecular mass markers (in kDa) are shown to the left (lane M). H and L respectively indicate the position of heavy and light chains of the IgG proteins. **(B) Reactivity and specificity of the purified UL51 antiserum analyzed by western blotting**. DEF were mock-infected (lanes 1 and 4) or infected with DEV CHv strain (lanes 2, 3, 5 and 6) and harvested at 24 h p.i. The pUL51 was separated by SDS-PAGE (lanes 1 to 3) and analyzed by western blotting using the UL51 antiserum (lanes 4 and 5) or the pre-immune serum (lane 6). Molecular mass markers (in kDa) are shown to the left (lane M). The arrowhead indicates the position of the pUL51 (about 34 kDa).

The purified UL51 antiserum and pre-immune serum was examined by SDS-PAGE (Fig 1A, lanes 1 and 2). To examine the reactivity and specificity of the UL51 antiserum, SDS-PAGE and western blotting was performed (Fig 1B, lanes 1 to 6). The results of western blotting showed that the UL51 antiserum reacted strongly with an approximate 34 kDa protein in lysates of DEV-infected cells (Fig 1B, lane 5). This band was not detected in mock-infected cells (Fig 1B, lane 4), and the pre-immune serum did not recognize any proteins in lysates of DEV-infected cells (Fig 1B, lane 6). These results indicated that the UL51 antiserum specifically detected the primary translation product of the UL51 gene; therefore, we used this UL51 antiserum for further experiments to study the locations of the DEV pUL51.

### Intracellular localization and distribution of DEV pUL51 in DEV-infected cells

**Figure 2:**
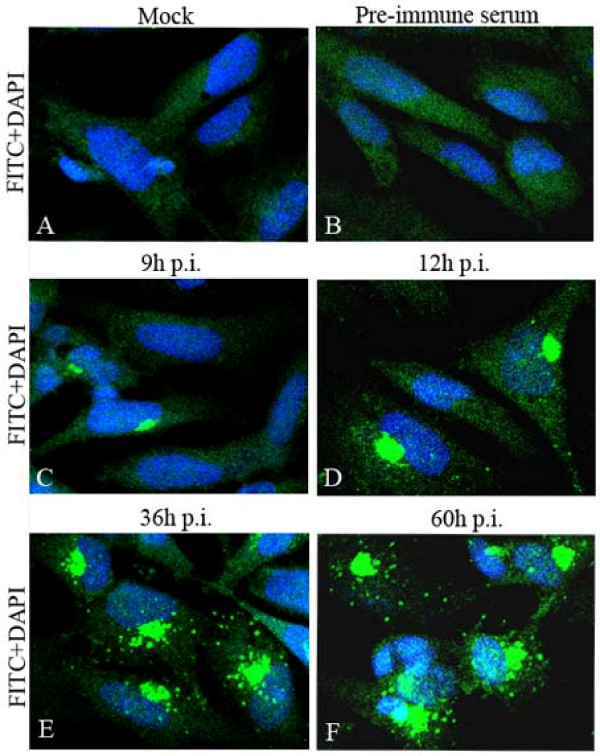
**Intracellular location and distribution of DEV pUL51 analyzed by IIF**. Mock-infected (A) and DEV-infected (B to F) DEF were fixed as described in Materials and Methods. The samples were stained with the UL51 antiserum (A, C to F) or pre-immune serum (B), and reacted with anti-rabbit IgG-conjugated FITC, and then counter-stained with DAPI (blue is representative the cell nuclei). The merged fluorescence microscopy images of DEF are shown in panels A to F with high magnification (600×).

A detailed analysis of the intracellular localization of DEV pUL51 was investigated using the purified UL51 antiserum or pre-immune serum by IIF staining of mock- and DEV-infected cells. As shown in Fig 2, a faint pUL51-specific fluorescence was first detected in the cytoplasm of DEV-infected cells at 9 h p.i. (Fig 2C), and then a strong fluorescence was observed mainly in the juxtanuclear region at 12 h p.i. (Fig 2D). After that, the pUL51-specific fluorescence in the juxtanuclear region was dense and localized on wide areas of the cytoplasm. At 36 h p.i. (Fig 2E), the pUL51-specific fluorescence was found widely distributed in the cytoplasm and especially was stronger in the juxtanuclear region; meanwhile, the nucleus of some DEV-infected cells also contained little fluorescence granular. Following by a series of morphological changes, the cytoplasm disintegration and nuclear fragmentation in DEV-infected cells, the intensity of the reaction increased at 48 and 60 h p.i. (Fig 2F), while the pUL51-specific fluorescence was mainly detected in the cytoplasm of infected cells and that one localized in the nuclear was faint. No pUL51-specific fluorescence could be detected in mock-infected cells reacted with the UL51 antiserum (Fig 2A) and in DEV-infected cells reacted with the pre-immune serum (Fig 2B).

### Subcellular localization and distribution of DEV pUL51 in DEV-infected cells

**Figure 3:**
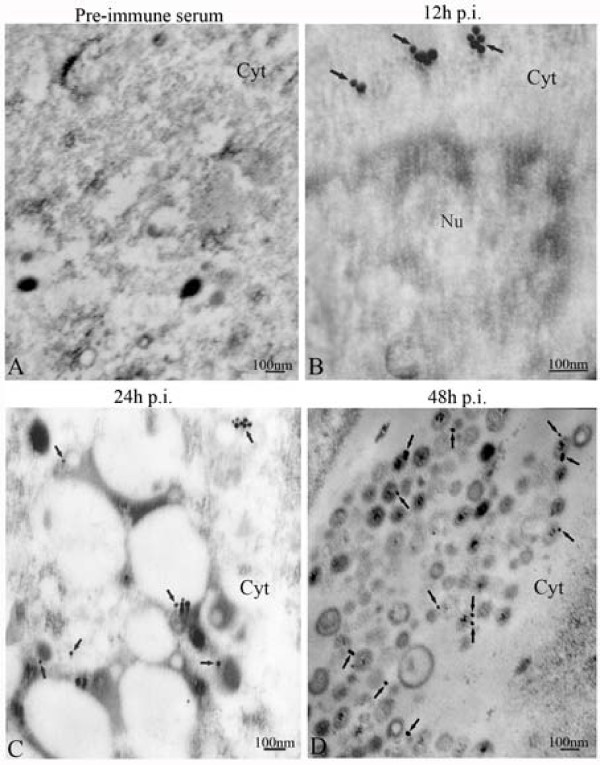
**Subcellular location and distribution of DEV pUL51 analyzed by TIEM**. Thin sections were prepared as described in Materials and Methods and incubated with the UL51 antiserum (B to D) or pre-immune serum (A) after treatment with 20% normal goat serum to block nonspecific antibody reactions. Samples were then incubated with anti-rabbit IgG-conjugated 10-nm-diameter gold particles. After extensive rinsing, sections were stained with uranyl acetate and lead citrate and examined with a Hitachi H600 transmission electron microscope at 75 kV. No specific immunolabeling was seen in the cells reacted with pre-immune serum (A). Immunolabelling for pUL51 (arrows) was found in the juxtanuclear region (B). Some immunolahelling for pUL51 (arrows) was found being associated with cytoplasmic virions and also with some membranous structure in the cytoplasmic vesicles (C and D). Abbreviations: Cyt, cytoplasm; Nu, nucleus. Bars represent 100 nm.

To identify the precise localization of DEV pUL51 in DEV-infected cells, TIEM was carried out. Immunoelectron microscopy showed that at 6 h p.i., only a little pUL51-specific immuno-labeling was first observed in the cytoplasm of DEV-infected cells (data not shown). At 12 h p.i., intense pUL51-specific immuno-labeling was detected in the juxtanuclear region (Fig. 3B), probably associated with Golgi apparatus. After that, ultrastructural changes of DEV-infected cells were especially remarkable, an increasing number of virus particles were accumulated in the cytoplasm with expansion of endoplasmic reticulum and formation of specialized vesicles. Starting from 24 h p.i., some immuno-labeling was found being associated with cytoplasmic virions and also with some membranous structure that was observed near the pUL51-specific immuno-labeling cytoplasmic virions in the cytoplasmic vesicles (Fig. 3C), and thereafter increasingly until 48 p.i. (Fig. 3D). At later times (60 h p.i.), the positive labeling was mainly localized in the cytoplasm and especially was scattered near the plasma membrane of DEV-infected cell (data not shown). No pUL51-specific immuno-labeling was seen in the DEV-infected cells reacted with the pre-immune serum (Fig. 3A) and in the mock-infected cells reacted with the UL51 antiserum (data not shown).

## Discussion

Proteins must be localized in the same sub-cellular compartment to co-operate towards a common biological function. The native subcellular localization of a protein is important for the understanding of its function [17]. However, due to the difficulties in the experimental determination of protein's cellular localization, the methods of theoretical prediction on the known sequence are becoming more important [26]. Computational tools provide fast and accurate localization predictions for any organism [17]. Generally, proteins are sorted into one of four localization classes: extra-cellular, cytoplasmic, nuclear and mitochondrial [27]. Our results of computer aided analysis revealed that the DEV pUL51 is not targeted to the mitochondrial, extra-cellular or nucleus, suggesting that it is targeted to the cytoplasmic in host cells, similar to the homologous proteins of HSV-1, BHV-1, and PrV [11,14,15].

Protein palmitoylation is one of the most ubiquitous post-translational modifications, reversibly attaching a 16-carbon saturated fatty acid as lipid palmitate to cysteine residues in protein substrates through thioester linkage. Also, palmitoylation is thought to be important in regulating intracellular trafficking, sorting, subcellular localization, protein-protein interactions, and functional activities of the proteins [28,29]. It has been reported that a number of viruses encode palmitoylated proteins, which play important roles in the process of virus replication [11,30-36]. In additional, previous reports have shown that palmitoylation of the N-terminal cysteine at position 9 (Cys-9) of the HSV-1 pUL51 is necessary for targeting to the Golgi apparatus [11]. From our results, one palmitoylation site is predicted at 9th cysteine of the DEV pUL51, suggesting that the pUL51 is also palmitoylation, and shares higher levels of homology with that of HSV-1. We thus inferred that palmitoylation at the N-terminal cysteine, which is conserved in all alphaherpesvirus UL51 homologs [15], is required for its membrane association and Golgi localization, although we cannot rule out the possibility that the pUL51 has another signal for its subcellular localization.

As we known, the Golgi Apparatus is an organelle central to the biosynthetic pathway of eukaryotic cells as it plays a principal role in the post-translational modification of newly synthesized proteins and in the sorting, packaging and distribution of these proteins to various destinations. To date, all the endogenous single membrane spanning proteins resident within the Golgi apparatus are Type II (cytoplasmic N-terminus/lumenal C-terminus) [19]. Our results of computer aided analysis indicated that the DEV pUL51 is also a Type II membrane protein, which contains a cytoplasmic N-terminus. Taking the above results, it is possible that the DEV pUL51 residents in the Golgi apparatus.

Furthermore, experimentally unravelling the native compartment of a protein also constitutes one step on the long way to determining its function [17]. Experimental determination of a protein subcellular localization is mainly accomplished by three approaches: cell fractionation, fluorescence microscopy and electron microscopy. Due to the cell fractionation approach is very sensitive to contaminations, we chose the fluorescence microscopy and electron microscopy approach to investigate the characteristics of pUL51 subcellular localization in this study.

Firstly, the results of IIF analyses revealed DEV pUL51 was found predominantly in the cytoplasm and especially in the juxtanuclear region, where they were detected as speckled or punctuate patterns in DEV-infected cells. These patterns are very similar to HSV-1 [11], BHV-1 [14] and PrV [15] pUL51 in viral infected cells. Moreover, Nozawa et al. reported that HSV-1 pUL51 localized to the juxtanuclear region, but only partially colocalized with the Golgi maker proteins such as the Golgi 58K protein and Golgi Matrix Protein (GM130) in HSV-1 infected cells [11]. Thus, combined with the mentioned above, we inferred that DEV pUL51 might remain mainly concentrated in the Golgi apparatus and ensures its incorporation into assembling virions.

Secondly, our TIEM analysis showed that an association of DEV pUL51-specific labeling with cytoplasmic virions and also with some membranous structure observed near the intracellular virion. Previous studies have reported that the HSV-1 pUL51 is eventually incorporated into virions and localized mainly to the inner side of cytoplasmic vesicles and/or the viral envelope in viral infected cells using protease digestion analysis [11]. These abservations suggested that the DEV pUL51 might be associated with viral envelopment in DEV-infected cells, and seemed to be incorporated into mature virions as a component of the tegurneut, similar to the HSV-1 pUL51.

Besides, it is reported that both proteins, HSV-1 UL11 and UL51, seem to contain specific Golgi-targeting signals, suggesting that both proteins might serve similar functions [15]. Recently, Loomis et al. reported that the tegument protein UL11 localizes to both the Golgi apparatus and the plasma membrane in HSV-1-infected cells [37]. Thus, like the HSV-1 UL11 protein, the DEV pUL51 also might efficiently accumulate in the Golgi apparatus at first, and then were sent to the plasma membrane from the Golgi by some unknown mechanism.

## Conclusion

In this study, we described the basic characteristics of pUL51 subcellular localization and distribution for the first time. From these results, we concluded that palmitoylation at the N-terminal cysteine, which is conserved in all alphaherpesvirus UL51 homologs, is required for its membrane association and Golgi localization, and the pUL51 mainly localized to the juxtanuclear region of DEV-infected cells, as well seemed to be incorporated into mature virions as a component of the tegument, consistent with its HSV-1 homolog UL51. The research will provide useful clues for DEV pUL51 functional analysis, and will be usefull for further understanding the localization properties of alphaherpesvirus UL51 homologs. Further studies will be aimed at constructing of the UL51 gene DEV mutant to study the function of the DEV pUL51.

## Competing interests

The authors declare that they have no competing interests.

## Authors' contributions

CJS, YFG, ACC and MSW carried out most of the experiments and drafted the manuscript. YZ, DL, HYX and NZ helped in experiments and drafted the manuscript. All authors read and approved the final manuscript.
